# Medical handovers: tacit consensus on interaction

**DOI:** 10.1007/s10459-025-10430-x

**Published:** 2025-04-14

**Authors:** Paulien Harms, Ninke Stukker, Tom Koole, Jaap Tulleken

**Affiliations:** 1 Department of Critical Care, University of Groningen, University Medical Center Groningen, Groningen, The Netherlands; 2 Center for Language and Cognition Groningen (CLCG), University of Groningen, Groningen, The Netherlands; 3 School of Human and Community Development, University of the Witwatersrand, Johannesburg, South Africa; 4 Wenckebach Simulation Center for Training, Education and Research, University of Groningen, University Medical Center Groningen, Groningen, The Netherlands

**Keywords:** Handover, Communication, Patient safety, Quality improvement, Discourse analysis

## Abstract

Recent studies on handover communication highlight the role of the incoming physician in preventing misunderstandings that contribute to medical errors. However, existing research often only provides abstract recommendations for increasing their participation, without specifying where and how this should occur. This paper applies discourse theory and methods to identify where the incoming physician’s active involvement is interactionally appropriate and can be integrated naturally and effectively. Twelve handovers between six pairs of resident physicians were recorded in a simulated ICU setting at a teaching hospital and analyzed using a combination of genre theory and conversation analysis. By first identifying the "moves" that constitute the handover genre, we pinpointed places where active participation by the incoming physician is expected and facilitates effective communication. While the *tasks and focus points* and the *questions and consultation* moves clearly invite such participation, the *clinical situation* move requires more negotiation, as the outgoing physician maintains control over the conversational floor, making it less immediately accessible for the incoming physician to contribute. The four remaining moves exhibit a more monologic pattern, where both participants display interactional behavior signaling that active input from the incoming physician is not anticipated. Our findings suggest that medical professionals share an implicit understanding of when participation is appropriate, shaped by conventions of the handover genre itself. By reconstructing these tacit rules through genre theory and conversation analysis, we provide insights that can inform training methods, ensuring that recommendations for active participation by the incoming physician align with the structured expectations of clinical practice.

**Supplementary Information:**

The online version contains supplementary material available at 10.1007/s10459-025-10430-x.

## Introduction

At the intensive care unit (ICU), at the end of a shift the responsibility and care for patients are transferred from a group of outgoing physicians to a group of incoming physicians. These handovers are an important step in ensuring continuity and quality of patient care. Unfortunately, communication failures during handovers, including miscommunication, frequently contribute to medical errors (Lingard et al., 2004; Greenberg et al., 2007; Arora et al., 2008; Horwitz et al., 2008; Bogenstätter et al., 2009; Pham et al., 2012; Robertson et al., 2014; Starmer et al., 2014).

In order to increase effectiveness of handover communication, earlier studies mostly focused on how outgoing physicians^1^ can organize handover information more effectively by providing standardized structures and mnemonic devices (e.g. SBAR, see Riesenberg et al., 2009). While these standardized structures undoubtedly contribute to more efficient and effective communication, they do not fully prevent misunderstandings. Importantly, existing standardized structures implicitly reinforce the prevailing belief that handovers are ‘sender driven’ treating them as a one-way transmission of information (Patterson & Wears, 2010; Wears & Perry, 2010). This perspective overlooks the role of the incoming physician, whose active participation in co-constructing understanding is increasingly recognized as essential (Nemeth et al., 2006; Wears & Perry, 2010; Manser & Foster, 2011; Berger et al., 2012; Cohen et al., 2012; Ilan et al., 2012; Flemming & Hübner, 2013; Manser et al., 2013; Rayo et al., 2014).

Despite growing recognition of the importance of the incoming physician’s role, active participation remains limited in current practice. The primary goal of the handover is the transfer of responsibility and patient care, which requires accurate information exchange. Since the incoming physician becomes the primary caretaker after the handover is done, the information given should be understood correctly for them to be prepared for their shift. Both physicians share responsibility in this process: the outgoing physician must present information in a way that can be understood, while the incoming physician must indicate when clarification or additional details are needed. In actual conversation, this understanding can be interpreted from their (non)verbal behavior - which will be explained in Sect. 3.4 below. Without these behaviors, it becomes more difficult to ensure the information transfer has been successful.

While these studies emphasize the need for more active participation from the incoming physician, it remains unclear how and when such participation is most effective. Rather than assuming that increased interaction is always beneficial, we argue that participation must align with the implicit structure of the handover itself. Our study reconstructs these implicit rules to identify where and when active involvement by the incoming physician is considered appropriate and interactionally relevant.

To do so, we use genre theory and conversation analysis to look at actual handovers in practice to reconstruct the implicit rules that make up the “handover genre”. Genres have their own set of rules that shape the communicative activity that it is. Genres are commonly viewed as frames, guiding every-day as well as professional communicative action. Understanding and mastering relevant genres is essential to professional success (Berkenkotter & Huckin, 1995; Trosborg, 2000). Learning professional genres often takes place implicitly, by observing and imitating expert professionals.

There are strong indications that making genre rules explicit and fostering genre awareness improves genre-related tasks. In literacy education for example, explicit instruction of genre conventions has been shown to enhance students’ writing performance (Bawarshi & Reiff, 2010; on medical professional writing specifically: Farooqi et al., 2022). To our knowledge not much research exists regarding effects of explicit instruction on performance of genres in interaction, but it is reasonable to expect similar positive effects (cf. Stukker et al., 2024).

Our aim in this article then, is to reconstruct the implicit rules of the medical handover genre, with an emphasis on the slots where active behavior of the incoming physician is deemed appropriate and relevant. Our analysis identifies options for promoting more active interactional behavior by incoming physicians in a natural way, respecting the tacit knowledge about handover genre rules among medical professionals. The results of our analysis will lay the groundwork for integrating active participation into medical handover training in a way that respects existing professional norms and tacit knowledge.

As we will see, our analysis of multiple handovers reveals consistent patterns in the interactive behavior of the incoming physician. During some parts of the handover the incoming physician’s responses are limited to more passive one-word tokens such as “yes” and “okay”. We will interpret this linguistic behavior as an indication that in these parts, initiating interaction is considered inappropriate, and thus: ineffective. In other parts, we find more elaborate listening strategies, such as asking questions. These we will interpret as indications that in these places, initiating interaction is considered appropriate and thus can be expected to lead to fruitful interaction between the outgoing physician and their incoming colleague.

## Theoretic framework

Genres are the ways in which people “get things done” using language (Paltridge, 2012: 62); journalists write news reports to inform the public about the latest news, people use manuals to construct cabinets and medical professionals perform handovers to transfer patients from one shift to another. Practicing a genre can be compared to playing a game, with its own rules and conventions (Bhatia, 1998: 25). Below we will discuss the central characteristics of “genre” as a phenomenon and explain why the medical handover is seen as one. A genre can be defined as a group of written or spoken discourses that share a common communicative goal that is achieved through recurring patterns regarding style, structure and, importantly, content. Discourse genres facilitate communication by establishing shared knowledge and standard practices within a community (Bhatia, 1993). In professional settings, genres allow for effective communication within disciplines such as law, education and journalism (Bakhtin, 1986; Bhatia, 1993; Trosborg, 2000) as well as medicine (Byrne et al., 1976; Drew & Heritage, 1992; Robinson, 2003).

A frequently cited definition of genre is Swales’ (1990):

*A genre comprises a class of communicative events, the members of which share some set of communicative purposes. These purposes are recognized by the expert members of the parent discourse community, and thereby constitute the rationale for the genre. This rationale shapes the schematic structure of the discourse and constrains choice of content and style (pp. 58).*

This definition highlights key elements relevant to medical handovers. The primary communicative purpose of a handover is to transfer information, responsibility and authority between physicians (e.g., Arora & Johnson, 2006; Cohen & Hilligoss, 2010 & Patterson & Wears, 2010). The internal structure of the handover consists of a specific order of what we call “moves”: stretches of text (or in our case often coinciding with “turns at talk”) serving a particular function in the genre (Upton & Cohen, 2009). For example, the handover typically starts with stating the* patient identifiers* before transitioning to a discussion of *the clinical situation * of the patient. This structured progression aligns with the way handovers are taught in medical education and training, since the aforementioned mnemonic devices also include similar moves as part of an “ideal” step-by-step structure.

A key aspect of genre theory is the role of expertise within a discourse community (Swales, 1990). In the context of medical handovers, experienced ICU staff members serve as role models, shaping how resident physicians learn to conduct these interactions (Passi et al., 2013). This reinforces Swales’ notion that communicative purposes are established and recognized by expert members of the discourse community. However, much of this expertise remains implicit, embedded in routine practice rather than explicitly articulated. Through our fine-grained analysis, we make these underlying interactional structures and genre conventions explicit, demonstrating how handover participants navigate and negotiate their roles within this communicative framework.

Another relevant component in Swales’ definition is the schematic structure of a genre which constrains content and style. It implies that the members of the discourse community are essentially not free to give any interactional contributions at any chosen time. This idea is central to our paper because it explains why interactive contributions of the incoming physician occur more frequently in certain parts of the handover than in others. It is particularly important to understand at what moments during the handover more active involvement from the incoming physician is permitted or even required, as it has implications for both handover training and patient safety.

Genre knowledge thus navigates participants through the overall structure of a discourse genre. At the same time, interactions still have to be managed locally. Interactions are constructed by participants taking turns at speaking (Hayashi, 2013). Who has or can take the conversational floor at any moment in the interaction is decided from moment to moment through an intricate system of turn-taking that is locally managed and party-administered (Sacks et al., 1974). Institutional interactions such as medical handovers are more pre-structured and predictable than casual conversations, often having to a certain extent predetermined notions on who may speak when, so-called “pre-allocation” (Sacks et al., 1974). Nonetheless, even with these “pre-allocated” turns, participants have some degree of freedom. Hence, local management and party-administration still play an important role in the accomplishment of turn transition at the local level (Hauser, 2009). In other words, from turn-to-turn participants still need to display what they are doing and where they are in the interaction. As we will see in the analysis, this becomes most evident from the handover participants’ behavior when they progress from one move to the next. We will interpret these linguistic signals of interaction organization as indications of places where interaction is considered appropriate or not appropriate.

## Methods

Our research team combined expertise from linguistics and clinical practice, ensuring an interdisciplinary approach that incorporated both interactional and medical perspectives. Researchers specialized in genre analysis and conversation analysis conducted the primary analysis, focusing on the structure and interactional dynamics of handovers. Additionally, the input from clinical experts was used for both data collection and to ensure that interpretations remained contextually relevant and correct in relation to actual medical practice.

### Participants

The 12 participants in this study included residents at different stages of their medical training, with varying levels of postgraduate clinical experience. Some were enrolled in specialty training (with an average of 36 months clinical experience), while others were not yet in a formal specialization program (with an average of 14 months clinical experience). Despite these differences in experience, all participants were familiar with the routine nature of handovers within their clinical practice. They worked in a hospital that operated on a night float system, consisting of three 8-hour shifts (day, evening, and night), meaning that they regularly engaged in structured handovers as part of their shift transitions.

Across the group, formal training in handover practices was largely absent. Most participants reported that they had developed their handover approaches informally through observation and practice in different clinical settings, a strategy known as implicit learning. This form of learning, where complex information is acquired incidentally without conscious awareness of what has been learned (Seger, 1994), is common in many professional contexts. While some participants had previously received brief instruction on the SBAR handover scheme, this was not common practice within the group.

### Data collection

The study was conducted at a teaching hospital in the Netherlands, where 12 simulated handovers with a duration ranging from 4 to 7 min were videotaped and analyzed. Each handover was performed by resident physicians in a simulated ICU environment, using documented cases of former ICU patients. The recordings were collected as part of a larger study on ICU shift-handovers.

For executing and recording the handovers, a vacant wing of the ICU was used. The simulation room was equipped with a patient manikin in a hospital bed, attached to medication pumps, intravenous fluids, and a computer monitor displaying the patient’s vital signs. Missing visual information, such as wounds, was provided through printed pictures.

The simulated cases closely reflect the way handovers are practiced in clinical training. In our hospital, as in many teaching hospitals, simulation-based exercises are a standard component of medical education, allowing physicians to rehearse and refine their communication and decision-making skills in a controlled environment. To enhance realism, the cases provided to participants were designed to mirror real ICU patient trajectories. Each case description was based on actual former ICU patients, ensuring that the content reflected real-world clinical complexities. The cases included not only the patient’s current medical status, but also relevant medical history, prior treatment decisions, and the course of hospitalization leading up to the handover. Both cases were representative of typical patients in a well-equipped, academic ICU in the Netherlands, ensuring the complexity level aligned with standard ICU practice.

The decision to use a simulated environment rather than the real ICU was twofold. First, handovers are inherently complex, involving multiple interacting variables such as case complexity, ambiguity and varying communication dynamics. To ensure the study remained both methodologically sound and practically feasible, we incorporated these key complexities while maintaining enough control to allow for systematic analysis. Without such control, occurring variation could obscure underlying interactional patterns. By structuring the simulated cases carefully, we aimed to reflect real-world variability while ensuring consistency across the data, making it possible to analyze handover communication in a controlled yet ecologically valid manner. Second, the use of a simulated setting helped to mitigate ethical concerns, avoiding the need to obtain consent from patients in critical condition or disrupt clinical workflows by introducing recording equipment into a high-stakes medical environment. While simulated, the handovers maintain relevance to real-world practice and provide valuable insights into the structure and interactional dynamics of medical handovers.

Although medical handovers follow a recognizable structure, individual instances can vary depending on factors such as the experience level of the participants, time constraints, and case complexity (Leenstra et al., 2018). These variations do not mean that each handover is entirely unique; rather, they occur within a shared communicative framework. By analyzing multiple handovers, we can identify prototypical characteristics that define shift-to-shift handovers as a genre while still accounting for contextual differences. This ensures that our findings reflect consistent patterns rather than isolated instances of variation.

### Procedure

Participants were recruited via email and asked to participate in pairs. They were informed that the study aimed to examine how handovers are enacted, what differences exist in the way they are conducted, and which practices are considered most effective.

Each participant performed two handovers: once as the outgoing physician and once as the incoming physician. The outgoing physician received a written description of the patient case, including lab results, echocardiograms, and other diagnostic information. Participants were given as much time as needed to process the information and prepare for the handover. To prevent copying of aspects of structure from the instruction text, the information was presented in a disorganized manner. Once the outgoing physician indicated they were ready, both participants were led into the simulation room to conduct the handover. The handovers were videotaped using three cameras: two at the corners of the foot of the bed and one at the head. This allowed the participants to move freely, while capturing every part of the interaction, both verbal and non-verbal (e.g., eye-gaze, nodding, deictic actions). The recordings were then compiled into a single frame and transcribed according to Jefferson’s transcription conventions (2004) (see Appendix A).

Immediately after each handover, participants independently completed a questionnaire assessing their shared *situation awareness* (SSA). The SSA questionnaire measured the extent to which team members collectively understood key informational elements of the case, interpreted them consistently, and aligned their expectations regarding future developments and practical implications (see also Endsley, 1995; Gardner et al., 2017). While these data are not the focus of the present analysis, the inclusion of this step is relevant for understanding how participants approached the handover task. By reflecting on the case details immediately afterward, participants demonstrated an orientation toward the handover as a meaningful and structured communicative activity, rather than as a purely artificial research task.

### Analytical approach

The central aim of this study is to determine where in the handover incoming physicians can become more actively involved. To answer this, we applied a two-step analytical approach that first identified the structural organization of handovers and then examined how participation was managed within these structures.

We began by examining the overall structure of the handovers through move analysis, which allowed us to identify the recurring communicative components within each handover. This step was necessary to determine which moves were primarily monologic, led by the outgoing physician, and which contained more interaction. However, move analysis alone does not provide insight into how participation is negotiated within these moves, nor does it reveal the specific interactional patterns that shape opportunities for engagement.

To gain more insight into these participation dynamics, we applied conversation analysis (CA) as our primary method. CA provides a microanalytical approach to studying interaction by examining both verbal and non-verbal behavior in detail. A core principle of CA is the idea that conversations are sequentially organized. Sequences can best be understood as a series of actions produced by interactants that achieve coordinated interactional activities (Schegloff, 1968, 2007; Schegloff & Sacks, 1973). Sequence organization revolves around the interdependence of adjacent turns of speech. This means that what a speaker does with one turn is contingent upon what has been done in a previous turn and sets up contingencies for what can be done in the next (Drew, 2013). For example, when one speaker asks a question, an answer - or at least an account for the absence of an answer– is expected to follow. Moreover, what a speaker does with a turn should be made recognizable for other participants; the design of their turn should reflect the action they perform.

By studying both the videotaped interactions and their transcriptions, which provide detailed representations of verbal and non-verbal conduct, we were able to systematically analyze turn-taking mechanisms, sequential structures, and participation alignment. This allowed us to examine how outgoing physicians structured their turns to shape interactional space and whether incoming physicians actively took opportunities to contribute. Through this approach, we identified specific moments in the handover where active participation by the incoming physician was either constrained or encouraged.

By combining move analysis with conversation analysis, we were able to pinpoint natural opportunities where a more active role for the incoming physician could be integrated without disrupting the structured flow of the handover. These findings provide insight into how structured participation patterns in handovers can inform training interventions, ensuring that increased participation is encouraged in a way that aligns with established communication practices in the ICU.

### Move analysis

Prior to this paper, we already identified the moves in our handover data, including their ordering. Move analysis consists of the systematic analysis of utterances or the combination of utterances (sequences) to determine their function in the interaction overall. The identification of moves was informed by existing literature on discourse genres and speech communities (Bhatia, 1993), as well as “practitioner advice, guide books, manuals etc. relevant to the speech community in question” (Bhatia, 1993, pp 23). For the coding of our moves, we used existing handover schemes and models such as SBAR (Riesenberg et al., 2009) as well as reviews of handover schemes and communication. Subsequently, we identified and, where necessary, specified the moves making use of content and linguistic information we encountered in the recorded handovers. The coding process was part of a larger project on analyzing handover structures and was completed in association with Leenstra and colleagues (Leenstra, 2020).

**Fig. 1:**
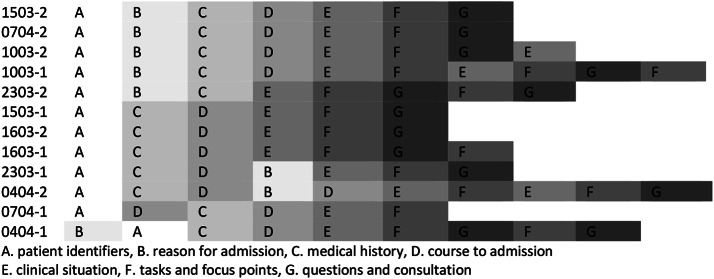
Ordering of moves by handover, in chronological order of similarity

The internal structure of the handover type we analyzed consists of seven moves. Although the specific ordering of these moves varied slightly across individual handovers– a limited amount of variation is a natural characteristic of genres (Swales, 1990; Bhatia, 1993) - we found consistency in the way participants progressed through them. The outgoing physicians usually start by giving (A) the *patient identifiers*, where they give the name and age of the patient. They either continue with (B) the *reason for admission* or advance directly to (C) the *medical history* of the patient. After the *medical history* we often find (D) the *course to admission*. The move in which (E) the *clinical situation* is discussed, is typically found at the center of the handover. Subsequently we find the move in which (F) the *tasks and focus points* are discussed and the handovers usually end with (G) the *questions and consultation*. Sometimes we would see a repetition of one of the moves. The most frequently observed sequence of these moves is presented in Fig. 1.

## Results

Our findings indicate that handover participants show consistent behavior in structuring handover communication, their interactional conduct reflecting an active orientation towards this organization. Furthermore, there is consistency with respect to the moves where active participation of the incoming physician is treated as appropriate. In the majority of moves, the outgoing physician serves as the primary speaker, while the incoming physician’s verbal contributions are scarce and typically limited to one-word responses such as “yes” or “hmhm”, indicating that they do not intend to contribute actively to the interaction.

Notably, all elaborate responses - where the incoming physician contributes beyond minimal acknowledgment tokens and actively engages in the conversation, for instance by asking questions or providing remarks - occur in only three moves: the *clinical situation*, the *tasks and focus points* and the *questions and consultation*.

The following section examines these patterns in detail. First, we demonstrate how, in specific moves, the behavior of both handover participants reflects an orientation to a primarily monologic structure limiting opportunities for the incoming physician’s participation, indicating that active interactional behavior on their part is not expected or desired. Second, we discuss evidence suggesting the three moves in which more elaborate responses occur, allow for more active participation by the incoming physician.

**Fig. 2:**
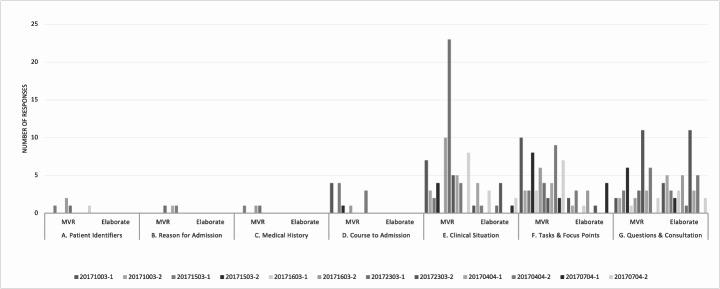
Response distribution of the incoming physician by move and handover

Figure 2 illustrates the distribution of responses by the incoming physician across different handovers. Each bar represents response behavior in an individual handover, with the Y axis displaying the number of responses by the incoming physician. Although behavior varies among individuals, the overall pattern suggests that interactional initiative by the incoming physician is largely absent in moves A-D, potentially including E, whereas it is appropriate in moves F-G.

### The participants orientation to the monologic nature of moves A-D

In our data, the outgoing physician takes up most of the speaking time, maintaining control over the so-called conversational floor. During the first four moves, the outgoing physician acts as the primary speaker, while the incoming physician’s verbal responses are scarce and limited to acknowledgment tokens. This shows that both physicians treat these moves as less appropriate– or even inappropriate– for active participation by the incoming physician.

A closer examination of the outgoing physicians’ interactional behavior reveals several recurring linguistic patterns that reinforce this asymmetry in participation. The outgoing physicians’ information delivery is structured in small chunks, with almost every new turn starting with a *filled pause* – typically the interjection “uh” or “uhm”, sometimes elongated (u::hm). Additionally, their utterances consistently end with a slightly rising intonation contour.

To illustrate how these features function in interaction we will take a look at an excerpt from the *medical history* and *course to admission* moves. This example demonstrates how the outgoing physician’s turn design signals their intent to maintain the conversational floor, while the incoming physician does not challenge this structure, reinforcing their orientation towards these parts of the handover as monologic. As we can see, the outgoing physician systematically starts their utterances with a filled pause – either “uh” or “uhm” – and frequently ends with a slightly rising intonation (marked by a comma at the end of the utterance) (See Excerpt 1).

**Excerpt 1:**
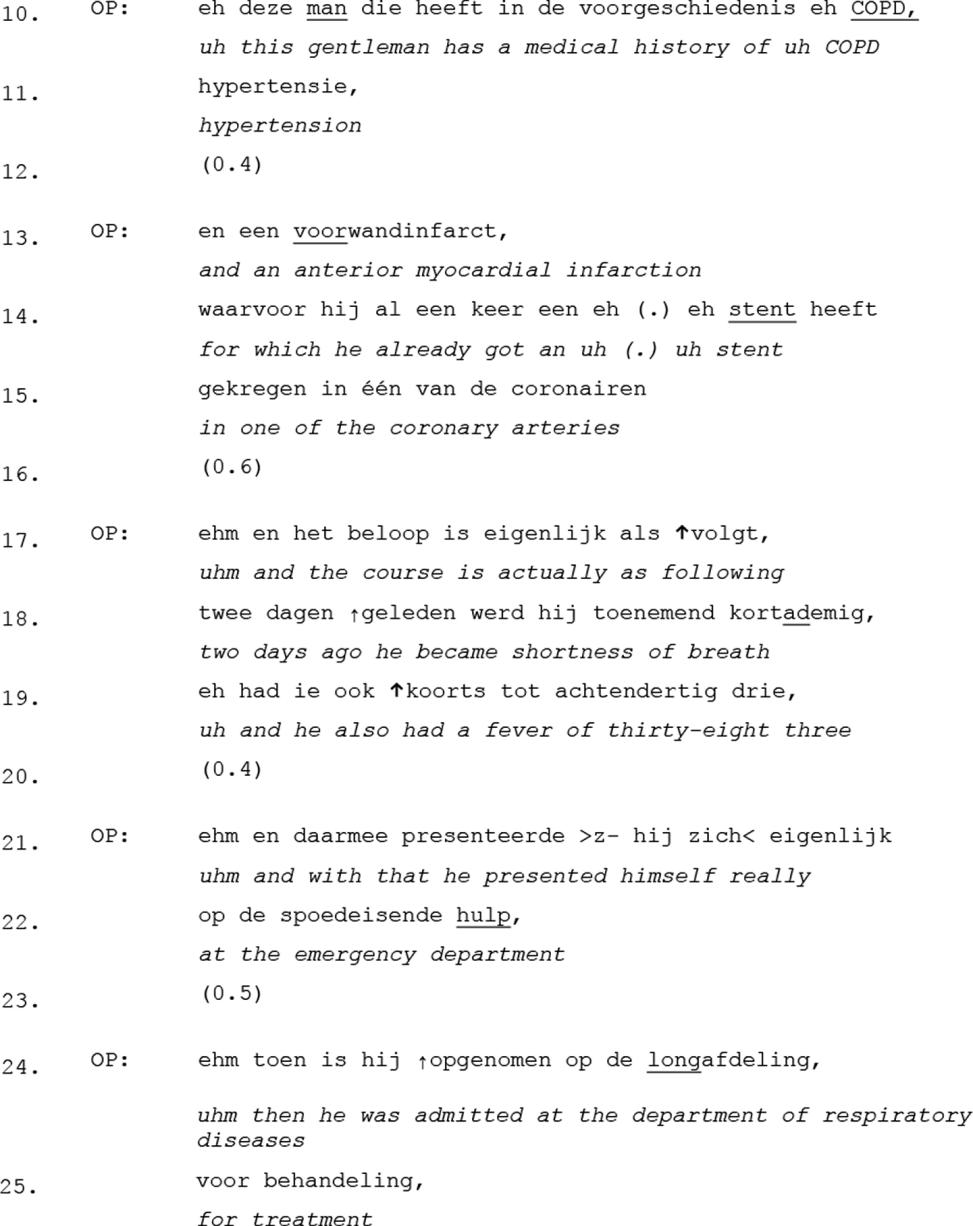
Transcript 20171003-2

As seen in the excerpt, each of the outgoing physician’s turns ends with a slightly rising intonation contour, while five out of the ten turns start with a filled pause (line 10, 17, 19, 21 and 24). In conversation analysis, filled pauses such as “uh” or “uhm” have been analyzed as a “turn-entry device”, effectively allowing speakers to maintain or claim control over the conversational floor (Sacks et al., 1974). Similarly, a constant, slightly rising intonation contour at the possible end of a turn is often understood as a signal that the speaker intends to keep the conversational floor (Selting, 2000). Together, these patterns create an interactional environment in which the outgoing physician structurally holds the conversational floor, limiting opportunities for active participation of the incoming physician.

Beyond these floor-keeping strategies, we do also see brief pauses between almost every unit of speech (line 12, 16, 20 and 23). These pauses could theoretically provide openings for the incoming physician to, for example, initiate a repair (i.e., asking for clarification) but no such attempts are made. Instead, the outgoing physician resumes speaking after each pause, reinforcing the pattern that this move of the handover is not treated as a space for co-construction. The absence of any attempt to take over the floor suggests that the incoming physician aligns with the outgoing physician’s structuring of the interaction, implicitly acknowledging that this is not the appropriate moment for active engagement.

Rather than solely functioning as floor-keeping strategies, filled pauses and slightly rising intonation contours play an active role in coordinating the sequential flow of the handover. These features help manage transitions between informational units while maintaining the expected structure of interaction. This highlights that a handover is not just about transferring information, but a coordinated process in which both physicians orient to shared expectations about when active participation is appropriate and when it is not.

In the previous paragraphs, we observed that although the incoming physician technically has opportunities to ask questions during the first four moves, they do not take them. This reinforces the idea that both parties share an understanding of the handover structure, recognizing that active participation by the incoming physician during these moves is not appropriate. While questions are absent, we do find another type of verbal response during these moves, namely *minimal response tokens* such as “yes”, “hmhm”, “okay” and “yes okay”. These vocalizations, commonly found throughout extended speech sequences, signal that the listener acknowledges the speaker’s turn and expects them to continue (Schegloff, 1982; Jefferson, 1984; Sacks, 1992; Gardner, 1997; Koole & Gosen, 2024). Unlike questions, which momentarily disrupt the ongoing turn to solicit additional information, minimal verbal responses are a non-interruptive form of “doing understanding”, reinforcing the outgoing physician’s control over the conversational floor and maintaining the flow of the handover.

In total the incoming physicians produced 206 minimal verbal responses without any additional verbal contribution (e.g., a question). Since minimal verbal responses typically occur in extended speech sequences and, in this case, represent the primary form of verbal engagement by the incoming physician during moves A-D, this suggests that the incoming physician treats these moves as handover segments led by the outgoing physician.

To illustrate this, we examine an excerpt from the *course to admission* move, where the only verbal responses from the incoming physician are the response tokens “yes” in line 27 and 31. This excerpt exemplifies how minimal responses function as non-disruptive acknowledgments, reinforcing the outgoings physician’s control over the interaction (See Excerpt 2).

**Excerpt 2:**
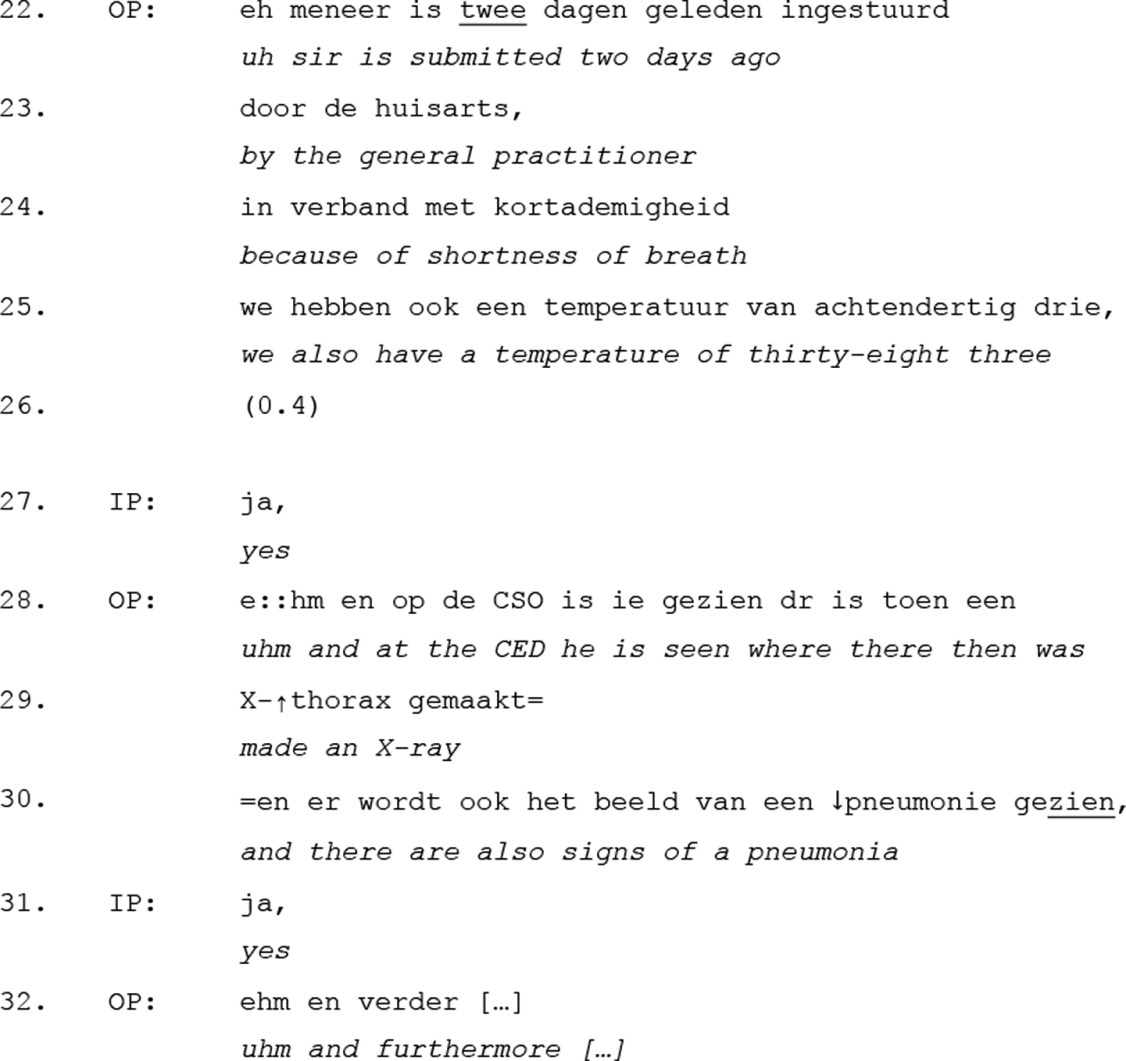
Transcript 20171503-1

This excerpt shows a clear example of how minimal response tokens – like the “yes” in line 27 and line 31 - function within moves A-D of the handover. First, these minimal responses typically function as acknowledgment tokens, signaling that the listener is following along without introducing a new conversational action (Schegloff, 1982; Jefferson, 1984; Sacks, 1992; Gardner, 1997). Rather than altering the course of the handover, they reinforce the expectation that the outgoing physician will continue speaking, a pattern further supported by their slightly rising intonation, which subtly indicates an orientation towards continued receipt of information. Secondly, their placement– both responses occur immediately after the introduction of new medical information (lines 25 and 30)– suggest that the incoming physician is confirming receipt rather than actively engaging. Unlike questions, which interrupt or redirect conversation, minimal responses are non-intrusive and affirm the outgoing physician’s control over the floor.

The concentration of minimal responses in moves A-D further indicates that this phase of the handover is primarily for information delivery rather than interaction. The incoming physician aligns with this structure by refraining from substantive contributions, using minimal responses instead to signal attentiveness without disrupting the flow. The use of minimal verbal responses by the incoming physician was also found during move E-G, but as we will see, during these moves we additionally find more elaborate responses, reflecting a change in the level of expected participation.

### Orientation to active participation in moves E - G

In contrast to moves A-D, where the incoming physician rarely displays active behavior and such participation does not seem to be expected by the outgoing physician, moves E-G show a shift toward more active participation from the incoming physician. The moves where we find most of the elaborate responses are the *clinical situation, tasks and focus points* and *questions and consultation.* Notably, two of these three moves occur at the end of the handover, and the interactional behavior of both participants clearly indicates that these are recognized as moments where active participation is appropriate.

The *questions and consultation* move consistently appears at the very end of the handover, and its name reflects the interactional behavior observed: this is where the incoming physician actively engages by asking for clarifications and seeking additional input. The *tasks and focus points* move, which always precedes the *questions and consultation move*, also invites active participation. Across the data, we identified two recurring patterns in how the transition between these two moves unfolds: (1) the outgoing physician explicitly prompts the incoming physicians by asking whether everything is clear or if there are any questions, or (2) the incoming physician takes the initiative, asking questions before the tasks and focus points move is fully completed, effectively initiating the transition to the last move. Both patterns were distributed equally across our data; occurring in six handovers each. The presence of these recurring structures suggests that, unlike in moves A-D, both participants treat these two moves as interactional spaces where active engagement from the incoming physician is expected and encouraged.

Excerpt 3 below illustrates the first pattern, in which the outgoing physician explicitly invites the incoming physician to ask questions, thereby initiating a question-answer sequence at the end of the handover. This transition occurs immediately following the *tasks and focus points* move, with the outgoing physician’s turn in line 123 marking the shift to the *questions and consultation* move.

**Excerpt 3:**
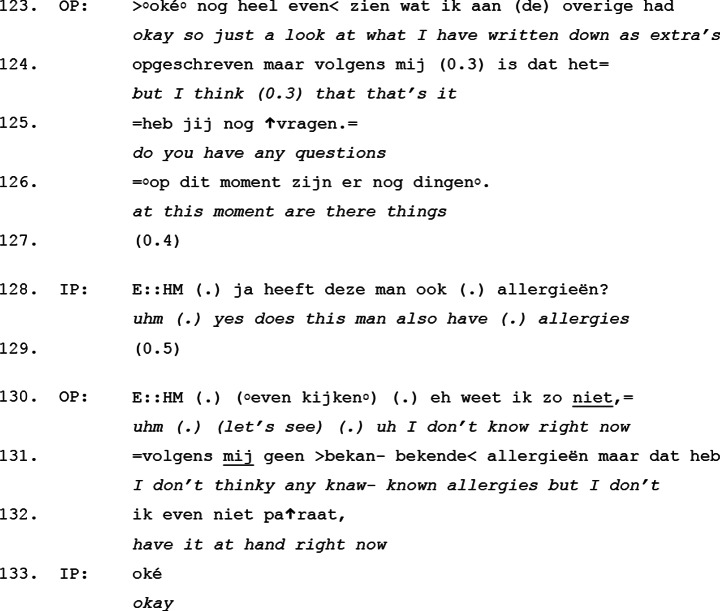
Transcript 20170404-1

This excerpt clearly demonstrates how the outgoing physician signals the transition from information delivery to the more interactive part of the handover. In lines 123–124, the outgoing physician explicitly closes the information-giving part of the handover by stating *“okay [I’ll] just have to check what I’ve written down for extra’s but I think that’s it”*. This turn acts as a pre-closing statement, signaling that no further information will be offered voluntarily. Immediately after this, in lines 125–126, the outgoing physician opens up the conversational floor for the incoming physician by asking *“do you have any questions, is there anything”*. This explicit question serves as an interactional cue, shifting the participation framework from a largely monologic format to a more dialogic one. The incoming physician responds in line 128, asking a question about the patient’s allergies, which is promptly addressed in lines 130–132. The answer gets accepted by a “sequence closing third” (Schegloff, 2007) in line 133 (okay). Notably, in many of our observed cases, the incoming physician initiates a new question-answer sequence after the initial one, demonstrating that once the conversational floor is explicitly opened, active participation becomes the norm rather than the exception.

In case of the second pattern, the outgoing physician does not explicitly invite the incoming physician to ask questions. Instead, the incoming physician independently initiates the transition to the *questions and consultation* move in response to information provided in the *tasks and focus points* move. The excerpt below illustrates how this transition unfolds as the incoming physician shifts the interaction towards the more dialogic part of the handover (See Excerpt 4).

**Excerpt 4:**
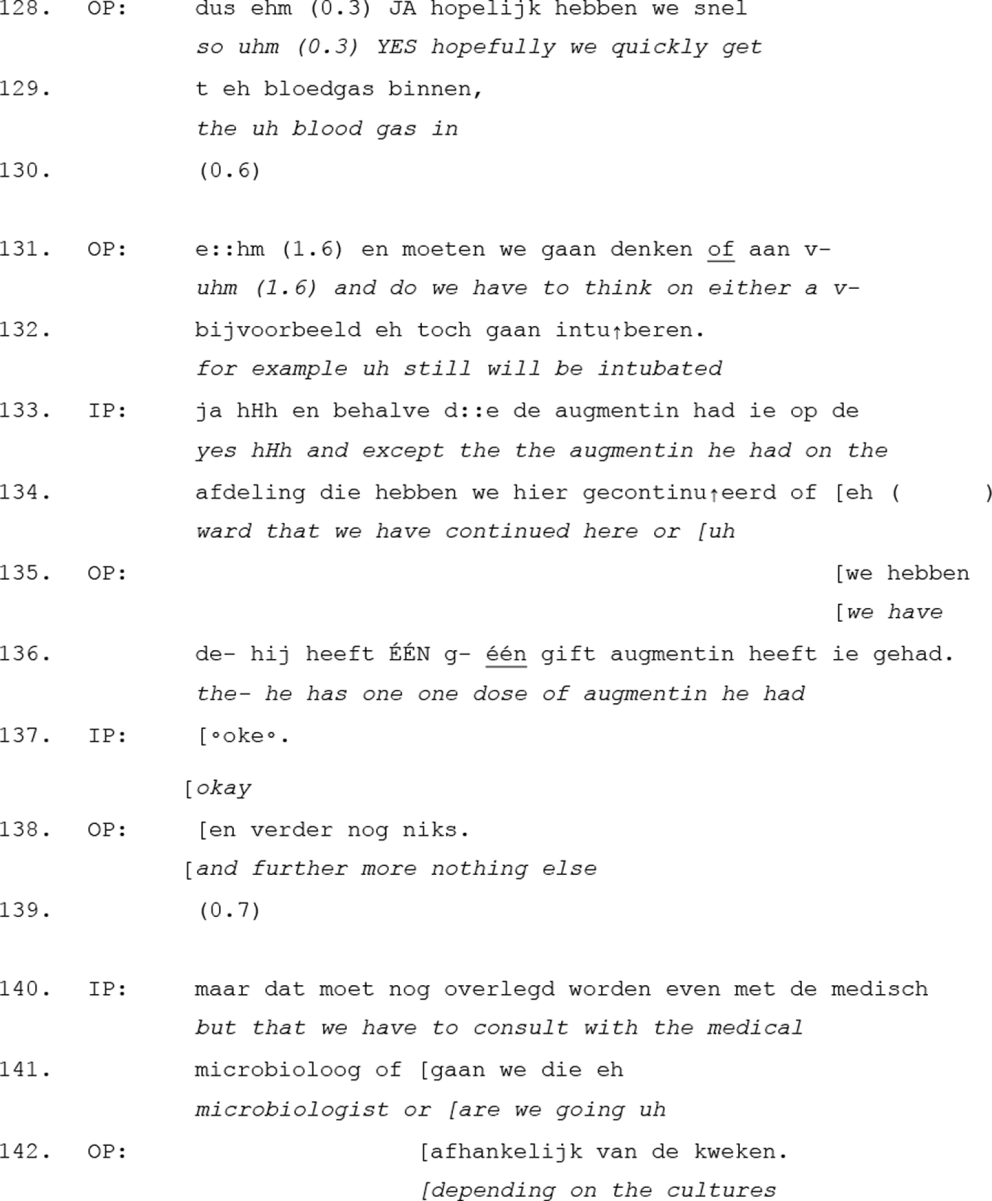
Transcript 20171503-1

Unlike the previous pattern, where the outgoing physician explicitly invites questions, here the incoming physician independently initiates the transition to the *questions and consultation* move. Instead of waiting for an explicit prompt, the incoming physician engages in the interaction at a moment that signals the end of information-giving, shifting the handover into a more interactive phase.

Prior to this excerpt, the outgoing physician summarized the most important information regarding the patient’s possible diagnosis. In lines 128–129 they then signal the possible conclusion of the information-giving part by stating that they hope the blood gas results will be available soon. The use of the adverb “so” (dus) at the beginning of this turn suggests some kind of rounding-off, summarizing prior information and implying that no further details are being added. This is reinforced in lines 131–132, where the outgoing physician makes a final treatment suggestion, speculating the need for intubation. At this point, rather than waiting for an explicit invitation, the incoming physician takes the initiative and asks a question about the current treatment of the patient (line 133–134). This marks the transition from the more monological part of the handover, to a more interactive one.

Notably, the outgoing physician does not resist this shift but immediately engages in the question-answer sequence, responding in lines 135–138. This confirms that both physicians orient to this part of the handover as the appropriate moment for interaction, even without an explicit prompt. The incoming physician further reinforces this orientation by asking a follow-up question in lines 140–141, which extends the discussion of the patient’s treatment plan.

Interestingly, the *questions and consultation* move is also the only move primarily organized/managed by the incoming physician. The outgoing physician does not attempt to close off this move - and thus the handover overall - as long as the incoming physician continues asking questions. Moreover, after a question has been asked and answered, we often observe longer pauses than in earlier parts of the handover. These longer pauses, exceeding the typical “beat of silence” between speaker turns (Jefferson, 1986; Selting, 2000), suggest post-completion of a speaking turn, creating an opportunity for another participant to take over the conversational floor. By not immediately continuing after answering a question, the outgoing physician implicitly provides space for the incoming physician to formulate additional questions, further reinforcing the interactive nature of this move.

This pattern suggests that genre knowledge plays a key role in structuring participation. Even though the outgoing physician does not explicitly open the floor for questions, the incoming physician recognizes that this phase of the handover provides the opportunity to engage. This demonstrates a shared understanding of the interactional structure, where the final moves of the handover are implicitly treated as spaces for discussion and clarification.

The only other move in which we find elaborate responses such as questions, is the move *clinical situation*. Unlike the *tasks and focus points* and the *questions and consultation* moves, the *clinical situation* move presents a more ambiguous interaction space. In 8 of the 12 handovers, the incoming physician gave some sort of elaborate response, however in 4 cases this was limited to a single instance. Most of these responses were requests for clarification or confirmation, rather than critical questions prompting a more extensive discussion.

Excerpt 5 illustrates how elaborate responses emerge in the move *clinical situation*, showing that the incoming physician’s participation is not explicitly invited but must be actively negotiated, indicating that this is not treated as a natural position for active interactional behavior by the incoming physician.

**Excerpt 5:**
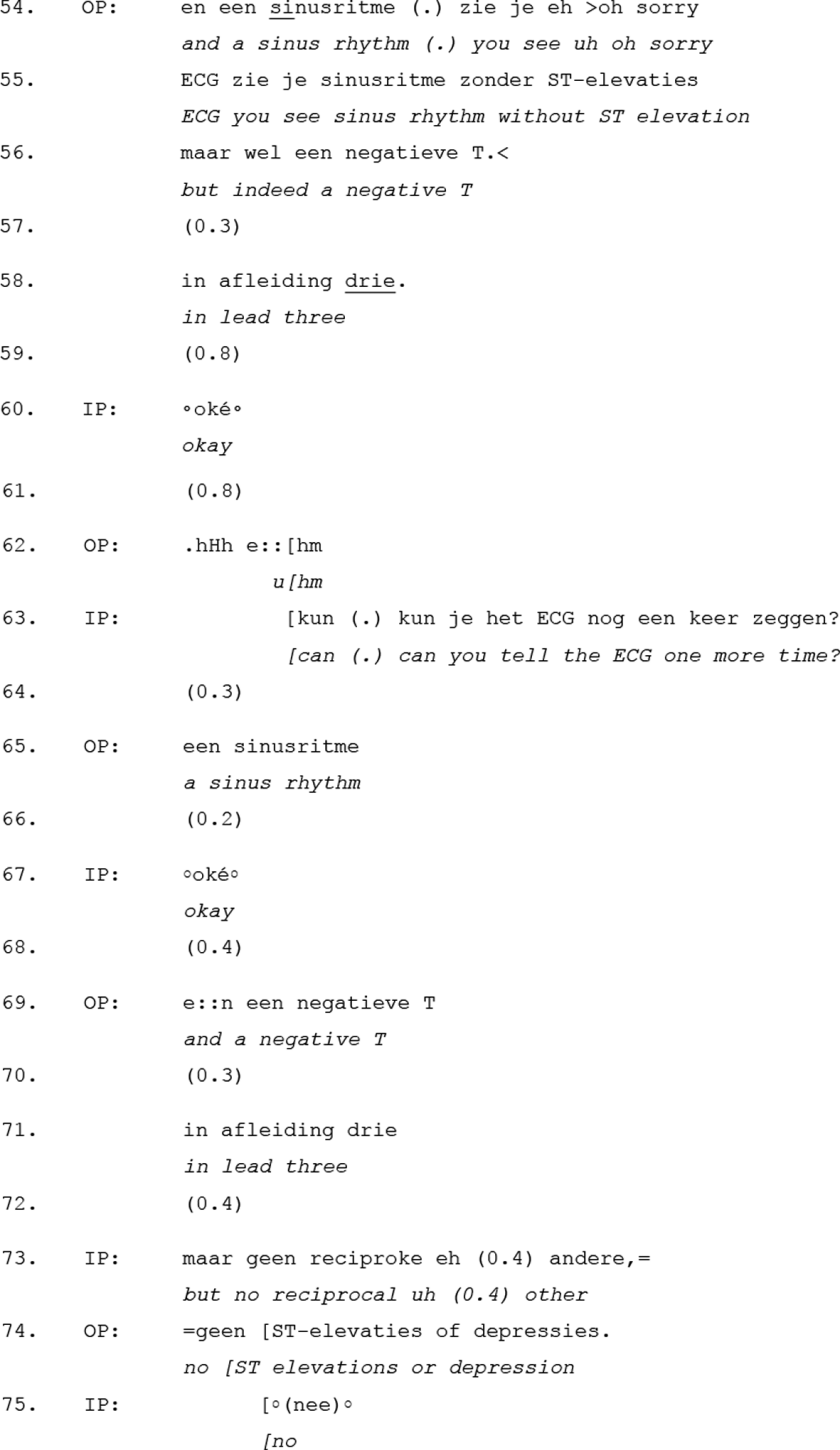
Transcript 20170704-2

This excerpt, taken from the middle of the *clinical situation* move, demonstrates how elaborate responses by the incoming physician require negotiation. Prior to this excerpt, the outgoing physician summarizes key aspects of the patient’s clinical status. In lines 54–58, they describe the ECG findings, following with a pause in line 59. Despite this potential transition space, the incoming physician initially responds with just a minimal response in line 60 (okay), followed by another pause in line 61, thereby not taking over the conversational floor.

The incoming physician does, however, eventually engage more actively in line 63 by requesting a repetition of the ECG findings. The timing of this turn entry is notable: rather than occurring during an available transition space (e.g., one of the previous pauses), it emerges as an interruption (indicated by the overlapping brackets in the transcript), forcing the incoming physician to assert participation rather than smoothly transitioning into the conversational floor. A likely explanation for this delayed response is that the incoming physician is actively taking notes throughout this part of the handover. The answer follows in line 65 to 71 by restating the ECG findings. This is followed by another clarification question in line 73, concerning reciprocal ST changes. The outgoing physician immediately provides a response in line 74, which is accepted by the incoming physician in line 75.

This pattern is consistently observed in the *clinical situation* move: responses by the incoming physician were often produced in overlap with, or directly “latched” to, an utterance of the outgoing physician rather than in clear transition spaces. While grammatically complete turns with falling intonation and pauses—such as in lines 54–58—signal possible turn transitions, the incoming physician does not take the floor at these points.

Similarly, after responding minimally with “okay” (line 60), the incoming physician remains silent for another 0.8 s, which would have been sufficient to initiate a more elaborate response. Instead, participation only occurs later, resulting in overlap in lines 62–63.

This suggests that during the move *clinical situation*, elaborate responses are not straightforwardly integrated into the interaction but have to be actively negotiated and the incoming physician here must assert engagement without clear structural cues for doing so.

This section highlights that most elaborate responses occur during the last two moves of the handover - the *tasks and focus points* and the *questions and consultation* - where active participation by the incoming physician is explicitly treated as appropriate. The only other move where such responses were observed is the *clinical situation*; however, in this move participation requires more negotiation.

## Discussion

This study taps into the growing recognition of handover interactions as co-constructed interactional accomplishments, emphasizing that the incoming physician plays a crucial role in shaping understanding. While existing literature largely views handovers as sender-driven, studies that do acknowledge the role of the incoming physician often lack specificity on how and when active participation should occur. This study lays the groundwork for specifying how the incoming physician could effectively take on a more active role by identifying the positions in handover communication where their contribution is treated as valuable. Our findings reveal that handovers are organized in accordance with a shared tacit understanding of the playing rules of the genre. Raising professionals’ awareness of discursive strategies, including the genre structures and interactional patterns we discussed, has proven effective in improving the quality and effectiveness of communication in professional and other settings where communication skills are crucial (Devitt, 2009; see also other contributions to Bazerman, 2009; Bawarshi & Reiff, 2010). The results of this study provide an instrument to add the role of the incoming physician to the picture in medical training.

Our findings indicate that questions and other forms of active engagement naturally emerge towards the end of the handover, where they are met with minimal resistance. In contrast, attempts at early engagement, as seen in the *clinical situation* phase, require more negotiation and may even disrupt the expected flow of the handover. Recognizing these pre-existing participation norms allows us to move beyond vague recommendations for “more active behavior” and instead focus on how participation can be effectively structured within the existing framework.

Given that our study was conducted in a simulated environment, we recognize the key differences between our data and real-life handovers. Factors such as time pressure, fatigue, and the presence of multiple team members in real ICU settings may introduce additional complexities that were not present in our study. However, despite these differences, our simulated handovers closely align with the way handovers are conducted in daily practice of the ICU setting in the hospital we studied. The structured nature of these interactions reflects the conventions that physicians internalize and apply in real practice. Furthermore, many of the interactional features we analyzed– such as filled pauses, intonation contours and turn-taking patterns– are deeply embedded in (spontaneous) speech and unlikely to be consciously simulated. This suggests that the conversational strategies observed in our data are not mere artifacts of the study design, but reflect genuine communicative behaviors in handovers. That said, real-world handovers are often more dynamic and complex than our simulated cases. Studying authentic handovers would allow for a deeper understanding of how contextual factors shape both structure and interaction, potentially explaining variations that deviate from the prototypical patterns identified in this study.

Our findings highlight a broader issue: should the pre-existing participation norms be accepted as given, or should handovers– and handover training methods– be restructured to facilitate a more receiver-driven model? If so, we must consider how the handover genre itself could be optimized to support more dynamic interaction throughout the entire process, rather than confining it to the final phases. One possible– though admittedly unconventional– approach could be to "invert" the structure of the handover, shifting responsibility for structuring the handover to the incoming physician. Instead of “passively” receiving information, the incoming physician could take the lead by posing questions, allowing the handover to unfold based on their informational needs rather than the outgoing physician’s predetermined structure. Such an approach could ensure that key concerns of the receiving physician are addressed early on, rather than being deferred to the final phases of the interaction.

This approach may also better align with the ways in which physicians interact with electronic patient records (EPRs). In modern hospital settings, incoming physicians are able to review patient data before the handover, meaning a receiver-driven handover could focus on clarifying, verifying, and contextualizing information rather than repeating known details. This shift could increase efficiency by reducing redundancy and ensuring handover time is used for decision-making and addressing gaps in understanding.

The current study laid the groundwork for a more sophisticated understanding of the interaction patterns that are tacitly maintained by medical professionals in handover communication. In future studies, we will refine the picture, focusing not only on the moves where active behavior by the incoming physician successfully occurs, but also on the communicative strategies they may use to successfully contribute to the handover (see Harms et al., manuscript in preparation). Ultimately, insights in current practice may be used to optimize handover communication in relation to the clinical task of handing over patient information effectively, minimizing the risk of medical errors due to misunderstanding or lack of information on the part of the incoming physician.

## Electronic supplementary material

Below is the link to the electronic supplementary material.

Supplementary Material 1

## Data Availability

Because of the privacy-sensitive nature of the data, it is not publicly accessible. However, the corresponding author can be contacted to discuss possible access upon request.

## Footnotes

1 Existing studies and papers regularly refer to the outgoing physician as the ‘sender’ and to the incoming physician as the ‘receiver’. Since the distinction between sender and receiver is treated differently in the field of communication, and a handover participant would take on both roles during one handover depending on the type of utterance they are performing (e.g. asking a question would make an incoming physician the sender of said question), we chose for the notion of ‘outgoing’ and ‘incoming physician’ which are less subject to interactional change.
