# Supplementary material for: Medical handovers: tacit consensus on interaction
Source: Adv Health Sci Educ Theory Pract. 2025 Apr 14;30(5):1641–63. doi: 10.1007/s10459-025-10430-x (PMC12572021; doi:10.1007/s10459-025-10430-x)
Supplement: Supplementary file 1 — Supplementary Material 1 [file 10459_2025_10430_MOESM1_ESM.docx]

**Appendix A: transcription conventions (adapted from Jefferson, 2004)**

| **Symbol** | **Explanation** |
| --- | --- |
| (0.5) | A pause lasting 0.5 s |
| (.) | A pause lasting 0.2 s or less |
| text1= =text2 | ‘Latching’: no observable silence between two adjacent turns-at-talk |
| [speaker 1  [speaker 2 | Overlapping talk |
| . | Falling intonation at the end of an utterance |
| , | Slightly rising intonation |
| ? | Strongly rising intonation at the end of an utterance* |
| ↑ | Upward shift in pitch |
| ↓ | Downward shift in pitch |
| word | Emphasis on underlined part of word/speech |
| wo:rd | Prolongation of the preceding sound |
| °text° | Pronounced relatively soft |
| TEXT | Pronounced relatively loud |
| te- | Speaker terminates production of a word or utterance |
| >text< | Pronounced at a higher speed than the surrounding talk |
| <text> | Pronounced at a lower speed than the surrounding talk |
| (text) | Unclear talk |
| (text) | Inaudible talk |
| *text* | English translation of the original Dutch text |

* When there is no symbol found at the end of an utterance, the intonation contour stays at the same level
